# Step-by-step combination of ^99m^Tc and ICG with endoscopic near infrared cameras in SLN mapping early-stage vulvar cancer

**DOI:** 10.1016/j.gore.2025.101677

**Published:** 2025-01-21

**Authors:** A. Rafael Guijarro-Campillo, Pablo Padilla-Iserte, Víctor Lago, Raquel Quintana-Bertó, Marta Arnáez-De La Cruz, Aníbal Nieto, Santiago Domingo Del Pozo

**Affiliations:** a Universitat de Valencia, Valencia, Spain; b Clinic Universitary Hospital Virgen de la Arrixaca, Murcia, Spain; c Universitary and Polytecnic Hospital La Fé, Valencia, Spain; d Instituto Murciano de Investigación Biomédica, IMIB, Spain

**Keywords:** Sentinel lymph node, Vulvar cancer

## Abstract

Vulvar cancer guidelines recommend inguinal sentinel lymph node (SLN) biopsy as the standard of care for patients with unifocal squamous cell carcinoma tumors less than 4 cm and clinically non-suspicious nodes in the groin [1]. The use of radioactive tracer is mandatory, while the application of blue dye is optional [2]. Combination detection techniques have been established as the most accurate for early vulvar cancer, with increasing evidence supporting indocyanine green (ICG) as an alternative to blue dye [3], [4]. However, protocols for ICG use remain heterogeneous, and the optimal protocol is yet to be defined [5], [6].

This video article presents a stepwise demonstration of the SLN mapping technique using a combination of radioactive 99 m-Tc and ICG with endoscopic near-infrared (NIR) cameras in two cases. A 52-year-old woman diagnosed with T1 vulvar cancer, with no extravulvar disease, was scheduled for wide local tumor excision and bilateral inguinal SLN biopsy. The procedure began with 99 m-Tc detection, followed by ICG identification. A 25 mg vial of ICG was dissolved in 10 mL of sterile water, with 2 mL injected into four intradermal quadrants around the tumor. Ten minutes post-injection, a small incision in the groin was made, assisted by lymphoscintigraphy fluorescence imaging using the NIR/ICG-IMAGE1S™ system. Images of another IB FIGO stage vulvar cancer patient undergoing SLN inguinal procedure with the NIR Da Vinci Xi camera were also included. The sentinel nodes were accurately detected in both patients, with no involvement after histological study. The informed consent for this video was obtained from both patients.

## Highlights

• The use of double labeling for SLN proceduce in vulvar cancer patients can make the technique more accurate and increase the detection rate.
• Despite current recommendations, there is no clear evidence and protocol on the use of ICG in sentinel node detection in early vulvar cancer.
• The use of ICG is feasible for the inguinal sentinel node technique in early vulvar cancer, and the use of NIR vision cameras that are commonly used in endoscopic gynecological surgery can be used for this purpose.

## CRediT authorship contribution statement

**A. Rafael Guijarro-Campillo:** Writing – review & editing, Writing – original draft, Methodology, Investigation, Conceptualization. **Pablo Padilla-Iserte:** Validation, Supervision, Methodology, Conceptualization. **Víctor Lago:** Validation, Supervision, Resources. **Raquel Quintana-Bertó:** Methodology, Formal analysis. **Marta Arnáez-De La Cruz:** Supervision. **Aníbal Nieto:** Resources, Conceptualization. **Santiago Domingo Del Pozo:** Validation, Supervision, Conceptualization.

## Declaration of competing interest

The authors declare that they have no known competing financial interests or personal relationships that could have appeared to influence the work reported in this paper.
